# The longitudinal impact of sports policy attitude on sport consumption intention among college students: a serial mediation of value internalization and exercise identity

**DOI:** 10.3389/fpsyg.2026.1828463

**Published:** 2026-06-04

**Authors:** Shuai Dong, Ruoyu Li, Zexi Zhao

**Affiliations:** 1 College of Basic Course, Shanxi Institute of Energy, Jinzhong, China; 2 Department of Physical Education, Jinzhong College of Information, Jinzhong, China; 3 The Department of Physical Education, Zhejiang University of Finance & Economics, Hangzhou, China

**Keywords:** exercise identity, longitudinal study, serial mediation, sport consumption intention, sports policy, value internalization

## Abstract

**Background:**

Within the macro-context of China’s “Sports Powerful Nation” strategy, governments and universities have invested substantially in promoting physical activity, yet the psychological mechanism linking macro-level sport policy to micro-level consumption behavior remains insufficiently understood. Drawing on Self-Determination Theory and Identity Theory, this study examines the longitudinal impact of sports policy attitude on sport consumption intention through value internalization and exercise identity.

**Methods:**

A three-wave longitudinal cross-lagged panel design was employed with 652 Chinese college students recruited from two universities in central and eastern China. To control for autoregressive effects and strengthen the basis for causal inference, all core variables were measured at Time 1, Time 2, and Time 3 (three-month intervals). Data were analyzed using structural equation modeling, and indirect effects were tested using bias-corrected bootstrapping (5,000 resamples).

**Results:**

After controlling for demographic covariates and baseline stability, T1 sports policy attitude significantly predicted T3 sport consumption intention (*β* = 0.076, *p* < 0.05). The relationship was serially mediated by T2 value internalization and T2 exercise identity, with the serial pathway accounting for the largest share (43.18%) of the total indirect effect.

**Conclusion:**

The translation of sport policy perception into sport consumption appears to involve a sequential psychological process from cognitive internalization to identity formation. The findings suggest that policymakers and university administrators may benefit from complementing value-promotion strategies with identity-based interventions to support sustained sport consumption among emerging adult consumers.

## 1 Introduction

In the contemporary landscape of global public health and economic development, the promotion of physical activity has evolved from a matter of personal well-being to a core component of national strategic governance (Liu et al., 2024; Song et al., 2025). Guided by frameworks such as the World Health Organization’s Global Action Plan on Physical Activity, governments worldwide are deploying macro-level policies to foster active societies (Shilton and Milton, 2024). Within this global paradigm, China’s “Sports Powerful Nation” strategy and the “National Fitness Program” represent a sustained institutional effort to systematically reshape the lifestyle of its populace (Yang et al., 2025). For college students—a demographic navigating the transition from late adolescence to early adulthood—these policies are designed not only to improve physical health but also to cultivate lifelong sport habits and to support the development of the sport economy (Yang et al., 2008). Despite substantial structural investments, however, a persistent implementation gap has been documented between top-down policy promulgation and bottom-up individual behavioral commitment (Chen et al., 2024; Downward et al., 2014). The question of how a macro-level institutional directive translates into a micro-level economic behavior—specifically, sport consumption intention—remains theoretically under-explained. Conventional economic-utility models offer limited explanatory leverage for understanding why students allocate their constrained discretionary income to sport-related goods and services, indicating a need to examine the psychological mechanisms that connect policy environments to consumption decisions.

The “Sports Powerful Nation” Strategy: Background and Campus Implementation. China’s contemporary sport policy architecture is anchored by two flagship initiatives. The Outline for Building a Leading Sporting Nation—commonly referred to as the “Sports Powerful Nation” strategy—was promulgated by the State Council in September 2019 and sets a long-horizon roadmap toward 2035. The Outline articulates five strategic tasks, including the cultivation of mass-fitness habits and the expansion of the sport industry to a target scale of five trillion RMB by 2035, together with eight implementation projects spanning youth sport, public-fitness infrastructure, and the sport-economy ecosystem. The complementary National Fitness Plan (2021–2025) operationalizes these goals at the population level by setting concrete targets for participation rates, per capita sport-venue area, and sport-related consumption expenditure.

On Chinese university campuses, these macro-strategies are implemented through several institutional mechanisms relevant to the present study. Mandatory physical education courses have been restructured under the Basic Standards for Physical Education in Higher Education Institutions, which require sustained sport engagement during the first 2 years of undergraduate study. The “One School, One Specialty” initiative encourages each institution to develop a signature sport culture, embedding sport identity within the campus environment. The Sunshine Sport Initiative and campus fitness-tracking systems further integrate physical activity into the broader student-development apparatus. These features make Chinese college students a theoretically informative population for examining how state-led sport policies are psychologically processed into market-relevant behaviors—the central question of the present study.

The initiation of behavioral transformation in this domain is rooted in the individual’s socio-cognitive appraisal of the surrounding environment. Drawing on Social Cognitive Theory, external environmental factors do not shape behavior directly but operate through cognitive processing (Bandura, 1986). In this context, sports policy attitude functions as the primary cognitive filter through which institutional signals are evaluated. When college students perceive national and campus sport policies positively—viewing them as supportive frameworks rather than coercive mandates—an autonomy-supportive psychological climate is established. Self-Determination Theory identifies such an environment as a precondition for value internalization (Deci, 1994), the organic process through which individuals transform socially endorsed goals into their personal belief systems (Deci, 1994; Stilz, 2017). A positive policy attitude facilitates the shift from extrinsic compliance to identified regulation, in which the health and social values of sport are autonomously endorsed (Jeanes et al., 2024). However, value internalization addresses why sport matters, and cognitive endorsement alone may lack the motivational intensity required to sustain higher-cost financial commitments. Recognizing the value of sport does not in itself imply willingness to purchase premium sport equipment or professional training services, suggesting that internalized value must crystallize into a more proximal psychological driver before consumption follows.

To bridge the gap between abstract values and concrete economic behavior, cognitive assimilation must extend into self-concept reconstruction. Identity Theory provides the theoretical architecture for this transition (Stets and Burke, 2000; Stryker, 1980). Behaviors tend to be most resilient and consistent when they are integrated into an individual’s self-definition. As students internalize the values advocated by sport policies, they may undergo a role-identity transformation, gradually adopting a salient exercise identity. Once a student conceptualizes themselves as an “exerciser” or “athlete,” sport consumption ceases to be a matter of functional necessity alone and increasingly becomes a vehicle for self-verification (Li and Qu, 2025). Self-verification theory holds that individuals are motivated to act in ways that confirm their established identities to themselves and to relevant others (Swann and Read, 1981). Sport consumption intention, on this account, is no longer a sporadic response to external stimuli but a stable, identity-signaling behavior. This cognition-to-identity evolutionary pathway provides a theoretical account of why identity may serve as a more durable anchor for long-term economic commitment than cognitive value alone.

Despite the theoretical coherence of this sequential mechanism, empirical validation has remained limited, primarily owing to methodological constraints in existing research. The majority of studies examining sport policy and consumer behavior have relied on cross-sectional designs, which do not capture the temporal precedence or psychological maturation inherent in the transition from environmental perception to identity formation. To address these gaps, the present study employs a three-wave longitudinal design to test a serial mediation model in which sports policy attitude sequentially influences sport consumption intention through value internalization and exercise identity. The study contributes to the literature in three respects. First, it provides longitudinal evidence consistent with a temporal trajectory of policy efficacy, complementing the predominantly cross-sectional evidence base in sport policy and sport consumer research. Second, it integrates Self-Determination Theory and Identity Theory to articulate the internalization–identification continuum underlying sport consumption, thereby extending the developmental logic of the Psychological Continuum Model (Funk et al., 2008; Funk et al., 2012) by anchoring its stages to specific psychological mechanisms. Third, it offers actionable implications for university administrators and sport marketers, suggesting that the cultivation of a sustainable sport economy depends not solely on promoting policy values but also on supporting identity-based engagement among emerging adult consumers.

## 2 Theoretical framework and hypotheses development

### 2.1 Bridging macro-policy and micro-consumption: insights from sport management literature

The psychological constructs central to this study—policy attitude, value internalization, identity, and consumption intention—are well established in general psychology, but their integration within sport management research remains underdeveloped. Sport policy scholarship has predominantly examined participation outcomes (Downward et al., 2014; Hoekman et al., 2017), with comparatively little attention to how policy environments shape the consumption behaviors that sustain the sport economy. The Psychological Continuum Model (PCM) advanced by Funk and James (2001) and elaborated by Funk et al. (2008) offers a foundational account of sport consumer behavior as a developmental progression from awareness through attraction and attachment to allegiance, with progressively greater investment of psychological and material resources at each stage. PCM-based research has, however, seldom incorporated macro-institutional antecedents such as policy perception, and has rarely modeled the underlying psychological maturation longitudinally. Sport identity research, in parallel, has documented robust associations between exercise or athletic identity and behavioral persistence (Anderson and Cychosz, 1994; Strachan et al., 2010), yet the upstream cognitive precursors that give rise to identity have remained theoretically under-specified within sport contexts. The present study contributes to this literature by modeling the cognitive-to-identity transition as a serial mechanism through which macro-policy environments translate into sustained sport consumption commitments among emerging adult consumers.

### 2.2 The direct relationship between policy attitude and consumption intention

**Figure 1:**
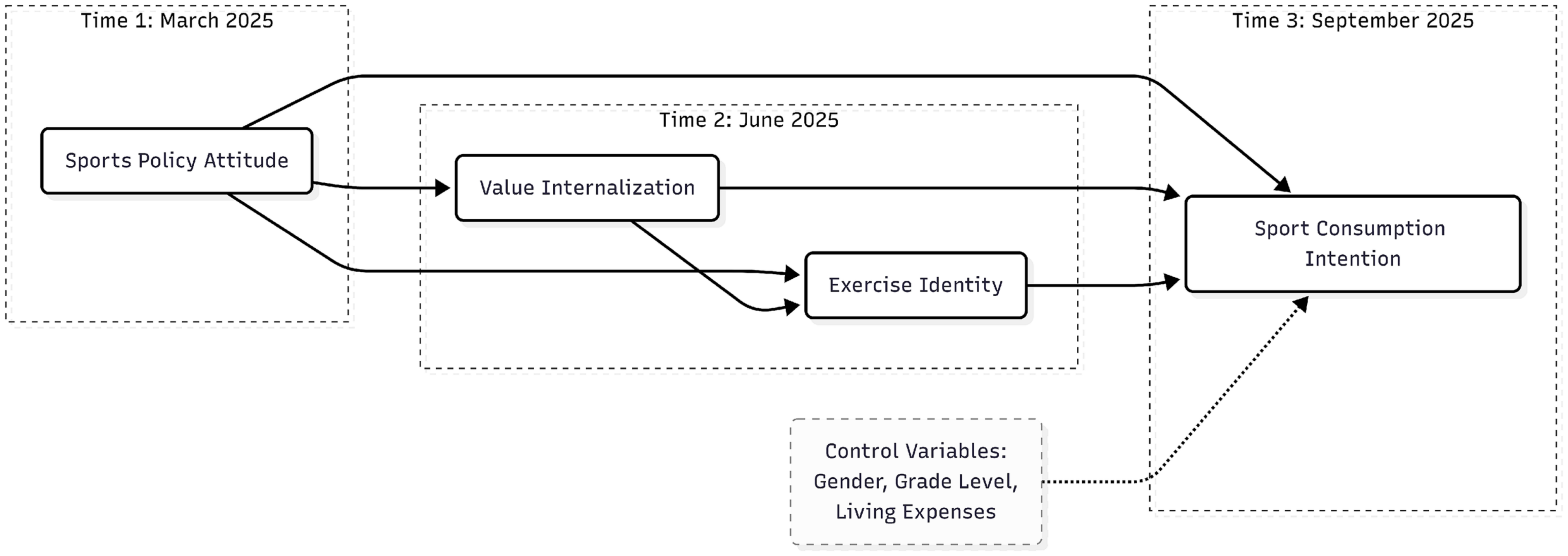
Conceptual model of the study.

The conceptualization of how macro-level environmental factors predict individual behavioral intentions is grounded in Social Cognitive Theory (Bandura, 1986) and ecological models of health behavior (Sallis et al., 2006). Within this framework, behavior emerges from continuous reciprocal interactions among cognitive, behavioral, and environmental determinants. In the sport context, sports policy attitude (T1) reflects an individual’s cognitive appraisal of the macro-institutional environment. A favorable attitude indicates that students perceive national and campus sports policies as supportive structural facilitators rather than restrictive mandates. Such an appraisal enhances perceived behavioral control and reduces perceived systemic barriers (Hagger and Chatzisarantis, 2014), thereby motivating the longitudinal allocation of discretionary resources—both temporal and financial—toward sport-related activities. Cognitive alignment with institutional support at T1 thus provides the foundation for sustained sport consumption intention at T3 (Figure 1).

Sport management research has examined the link between institutional environments and sport behavior primarily through ecological and policy-perception frameworks. Sotiriadou et al. (2014) demonstrated that perceived policy support and stakeholder engagement shape sport participation pathways at the attraction and retention stages, while Wicker et al. (2013) showed in multi-level analyses that sport infrastructure availability—a tangible expression of public sport policy—significantly predicts individual sport participation. More recent work on Chinese student populations has differentiated compliance-driven from autonomy-supportive policy perceptions, with the latter consistently emerging as a stronger predictor of voluntary, discretionary sport behaviors (Song et al., 2024). Chinese college students, situated at the intersection of state-led sport mobilization and an expanding consumer market, represent a theoretically informative population for examining this process, yet the longitudinal translation of policy attitudes into market-relevant intentions has received little empirical scrutiny.

*H*_1_: College students’ sports policy attitude at T1 has a significant positive longitudinal effect on their sport consumption intention at T3.

### 2.3 The mediating role of value internalization

The mechanism through which external policy environments translate into internal motivation is articulated by Self-Determination Theory (Deci and Ryan, 2000), and more specifically by its Organismic Integration Theory (OIT) sub-framework. OIT posits that individuals internalize external regulations to the extent that the surrounding social context is perceived as autonomy-supportive rather than controlling. A favorable sports policy attitude at T1 functions as such an autonomy-supportive context, facilitating value internalization at T2 and enabling the transition from extrinsic to identified regulation. When the value of an activity becomes internalized, it is integrated into the individual’s personal belief system, so that subsequent engagement is sustained by personal significance rather than by transient external rewards (Figure 1).

Although SDT has been extensively applied to exercise behavior, with autonomous forms of regulation consistently predicting participation and adherence (Teixeira et al., 2012), its application to sport consumption has emerged more recently. Funk et al. (2012) explicitly extended SDT to sport consumer research, demonstrating that autonomy- versus control-oriented motivation differentially regulates the engagement of sport consumers, with autonomously motivated consumers exhibiting stronger and more durable behavioral commitments. Building on this work, the present study reasons that when policy environments enable students to autonomously endorse the value of sport, this internalization functions as a psychological warrant for discretionary sport-related expenditure even under the financial constraints typical of college life.

*H*_2_: Value internalization at T2 mediates the relationship between sports policy attitude at T1 and sport consumption intention at T3.

### 2.4 The mediating role of exercise identity

Whereas value internalization captures the cognitive evaluation of sport, Identity Theory (Stets and Burke, 2000; Stryker, 1980) provides the conceptual lens for understanding the self-concept stabilization required for persistent economic behavior. Exercise identity at T2 is defined as the extent to which the role of “exerciser” or “athlete” is incorporated into an individual’s self-concept (Anderson and Cychosz, 1994). A supportive sports policy attitude at T1 fosters a salient community of practice on campus that encourages adoption of the exerciser role through social categorization. Once this identity is established, sport consumption transcends functional utility and becomes a mechanism for self-verification (Oyserman, 2009). Individuals with a strong exercise identity exhibit higher intentions to acquire sport equipment, apparel, and services, as these consumption behaviors function as identity-signaling acts that validate the self-concept to the individual and to relevant others. Because identity occupies a more central position in the self than transient attitudes, it constitutes a stable psychological anchor for longitudinal consumption intentions (Figure 1).

Identity-related perspectives have gained traction in sport consumer research. Beaton et al. (2011), in a large-scale empirical analysis of marathon runners (*N* = 3,117), demonstrated that the symbolic value and centrality of sport involvement—both core indicators of identity-laden engagement—differentiate consumers along the developmental stages of the PCM and predict the frequency, depth, and breadth of sport-related behaviors. This contribution has, however, largely treated exercise identity as a stable individual difference rather than as a dynamic outcome of policy-induced value internalization. The present study addresses this gap by modeling identity formation as an endogenous process embedded within the broader policy-to-consumption trajectory.

*H*_3_: Exercise identity at T2 mediates the relationship between sports policy attitude at T1 and sport consumption intention at T3.

### 2.5 The chain mediating effect

To articulate the psychological process linking policy perception to consumption intention, this study integrates SDT and Identity Theory to specify a sequential cognition-to-identity pathway. Value internalization and exercise identity are theorized to operate not as parallel mediators but as a serial mediating chain. A positive policy attitude at T1 first functions as an environmental catalyst that promotes the internalization of sport-related values at T2. This cognitive endorsement subsequently provides the ideological foundation for identity construction at T2, as it is psychologically inconsistent to develop a salient identity around an activity that the individual does not fundamentally value. The crystallized exercise identity then operates as the proximal driver of sustained sport consumption intention at T3. The proposed pathway thus delineates a coherent trajectory from environmental perception (attitude), to cognitive assimilation (value), to self-concept reconstruction (identity), and ultimately to behavioral commitment (intention; Figure 1).

The integration of motivational and identity perspectives has remained rare in sport consumption research. Existing work has typically examined SDT-based motivation (Funk et al., 2012; Teixeira et al., 2012) and exercise or sport identity (Beaton et al., 2011; Strachan et al., 2010) within separate streams, without articulating a temporal sequence between value internalization and identity formation. By specifying a longitudinal serial pathway, the present study advances sport management theory in three respects: it repositions exercise identity as a consequence of, rather than a substitute for, autonomous internalization; it extends PCM logic by anchoring its developmental stages to the specific psychological mechanisms posited by SDT and Identity Theory; and it provides a theoretically explicit account of why certain policy interventions succeed in cultivating active sport consumers while others stall at the awareness stage.

*H*_4_: Value internalization and exercise identity at T2 play a serial mediating role in the longitudinal relationship between sports policy attitude at T1 and sport consumption intention at T3.

## 3 Method

### 3.1 Participants and procedure

To capture the dynamic psychological mechanism through which macro-policy perception translates into micro-consumption behavior, this study employed a three-wave cross-lagged panel design spanning 6 months (from March 2025 to September 2025). To ensure sample diversity and enhance the external validity of the findings, a stratified cluster sampling method was utilized to recruit participants from two distinct types of higher education institutions in China. Specifically, the sample included students from the Shanxi Institute of Energy (representing STEM-focused universities in central, developing regions, characterized by pragmatic orientations) and Zhejiang University of Finance & Economics (representing business-focused universities in eastern, highly developed coastal regions, characterized by high market sensitivity). This cross-regional and cross-disciplinary sampling strategy helps reduce the possibility that the findings are attributable to a single regional economic context or institutional culture of a single macroeconomic environment or institutional culture on sport consumption intentions.

Prior to data collection, the research protocol was reviewed and approved by the Institutional Review Board (IRB). Informed consent was obtained from all participants, with strict assurances regarding voluntariness, confidentiality, and data protection. To accurately match longitudinal responses while maximizing privacy protection, this study utilized the advanced tracking features of a professional online survey platform. At Time 1, participants registered using their student IDs, which the system’s backend automatically converted into a one-way encrypted virtual identification code (Hash ID). During the Time 2 and Time 3 follow-ups, the system distributed the surveys via targeted, token-based links. Researchers only had access to the anonymized Hash IDs upon data export. This double-blind design ensures precise multi-wave data matching while completely circumventing the memory errors and privacy risks associated with traditional self-generated identification codes.

The data collection proceeded in three distinct phases to construct a robust Autoregressive Cross-Lagged Panel Model (CLPM). To accurately capture the dynamic changes and control for historical inertia (autoregressive effects), all four core variables—Sports Policy Attitude, Value Internalization, Exercise Identity, and Sport Consumption Intention—were repeatedly measured at each of the three time points:

- Time 1 (March 2025): At the commencement of the spring semester, the baseline survey was administered to assess demographic covariates along with the first wave of the four core variables. A total of 850 questionnaires were distributed. After embedding attention check items (e.g., “Please select ‘Strongly Agree’ for this item”) and excluding invalid responses (e.g., completion time under 120 s, straight-lining), 812 valid responses were retained.
- Time 2 (June 2025): 3 months later, 2 weeks prior to the final examination period (to avoid stress-induced psychological confounding), the 812 participants from T1 were re-contacted via targeted links to complete the second wave of measurements for all four core variables. Due to internships, leaves of absence, or non-responses, 745 valid questionnaires were successfully matched.
- Time 3 (September 2025): After another three-month interval, at the beginning of the fall semester—a peak period for students to allocate their semester budgets—the remaining 745 participants were surveyed to complete the final wave of measurements for the four core variables. Following rigorous multi-wave matching and missing data screening, a final valid sample of *N* = 652 was obtained, yielding an effective longitudinal retention rate of 76.7%.

**Figure 2:**
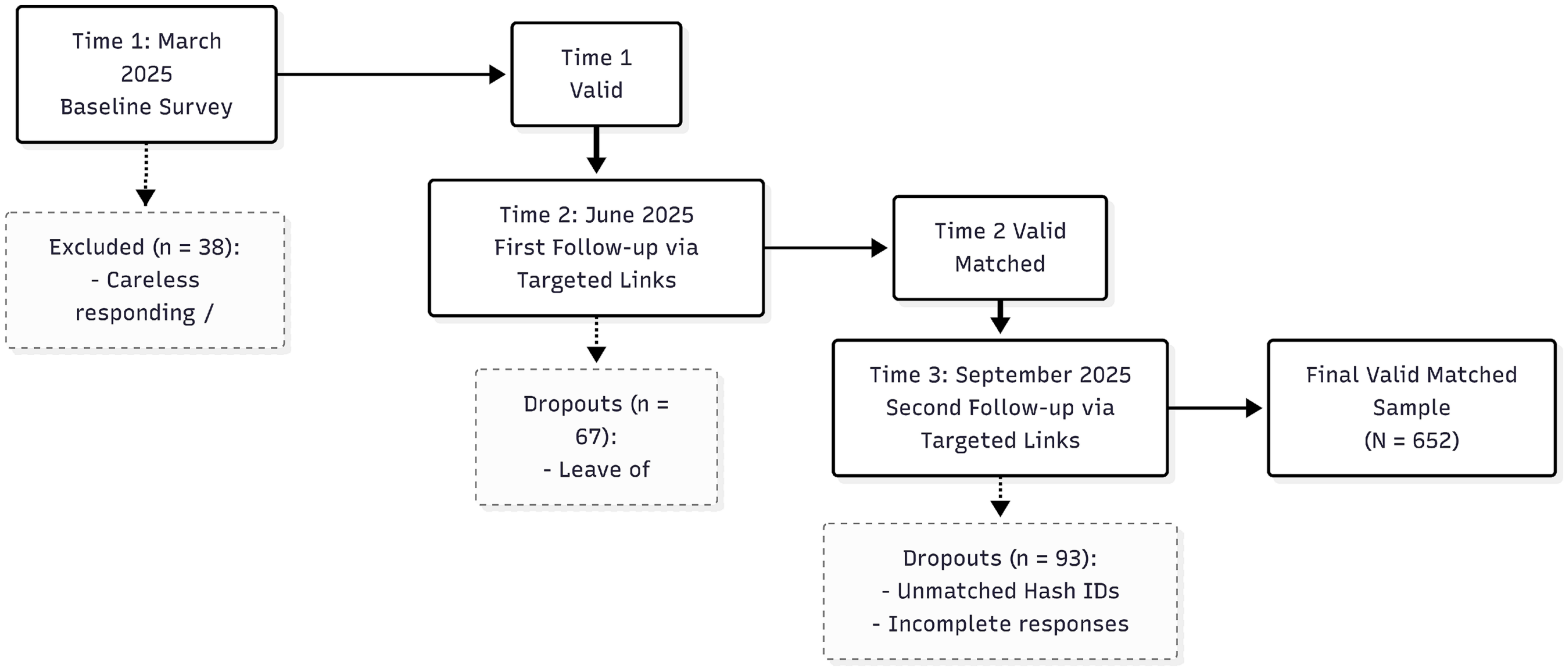
Longitudinal study data collection and sample attrition flowchart.

The final sample consisted of 345 males (52.9%) and 307 females (47.1%), with 318 students (48.8%) from the Shanxi Institute of Energy and 334 students (51.2%) from Zhejiang University of Finance & Economics. The mean age was 19.85 years (*SD* = 1.12, range = 17–24 years; Figure 2).

Attrition Analysis: To rule out “survivorship bias”—the possibility that only individuals highly interested in sports completed all three waves—independent samples t-tests and chi-square tests were conducted to compare the baseline characteristics of the retained sample (*N* = 652) and the dropouts (*N* = 198). The results indicated no statistically significant differences between the two groups regarding T1 Policy Attitude (*t* = 0.84, *p* = 0.402), age (*t* = −0.56, *p* = 0.576), and gender distribution (*χ^2^* = 1.15, *p* = 0.283). These results suggest that attrition was unlikely to be systematically associated with the observed baseline characteristics, although MCAR cannot be definitively established.

### 3.2 Measures

All psychological and behavioral constructs in this study were measured using established scales from existing literature. To ensure semantic equivalence and content validity within the Chinese context, a rigorous translation and back-translation procedure was executed, followed by face validity assessments by two psychology professors and one sports economics expert. All items were rated on a 5-point Likert scale ranging from 1 (Strongly Disagree) to 5 (Strongly Agree). Higher scores denote higher levels of the respective construct.

#### 3.2.1 Sports policy attitude (T1)

Adapted from the framework by Song et al. (2024) and contextualized according to China’s “Sports Powerful Nation” directives. This 6-item scale captures two core dimensions: Policy Awareness and Perceived Support. A sample item is: “I believe the current campus sports policies are not merely mandatory requirements, but provide strong support for my physical and mental development.” In the current study, the scale demonstrated good fit in the Confirmatory Factor Analysis (CFA), with a Cronbach’s *α* of 0.88 and a Composite Reliability (CR) of 0.89.

#### 3.2.2 Value internalization (T2)

Grounded in the Organismic Integration Theory (OIT) sub-framework of Self-Determination Theory (Deci and Ryan, 2000), value internalization was operationalized using the four-item Identified Regulation subscale of the Behavioral Regulation in Exercise Questionnaire (Markland and Tobin, 2004). OIT conceptualizes internalization as a continuum spanning external regulation, introjection, identification, and integration, and the present study selected the Identified Regulation subscale rather than a composite of all regulatory styles for three substantive reasons. First, identified regulation marks the pivotal autonomous threshold at which the personal value of an activity is consciously endorsed, and meta-analytic evidence indicates that it is the strongest motivational correlate of sustained exercise behavior (Teixeira et al., 2012). Second, the theoretical model of the present study concerns the cognitive endorsement of sport-related values rather than guilt-driven (introjected) or fully assimilated trait-level (integrated) regulation; identified regulation therefore most directly maps onto the construct of interest. Third, integrated regulation typically requires longer developmental periods than the six-month observation window of the present study to fully consolidate (Wilson et al., 2006), making it less appropriate for a college-aged sample. Sample items include “I participate in sports because I value the health benefits it brings” and “I think it is important to make the effort to exercise regularly.” The Cronbach’s *α* for this subscale was 0.86, with a composite reliability (CR) of 0.85.

#### 3.2.3 Exercise identity (T2)

Assessed using the well-established Exercise Identity Scale (EIS) developed by Anderson and Cychosz (1994). To suit the longitudinal tracking context and reduce respondent burden, nine highly representative items were retained, capturing Role Identity (e.g., “I consider myself an exerciser”) and Exercise Beliefs (e.g., “I would feel a real loss if I were forced to give up exercising”). This scale exhibited excellent reliability, with a Cronbach’s *α* of 0.91 and a CR of 0.92.

#### 3.2.4 Sport consumption intention (T3)

Adapted from the sport consumer behavior scale by Lagoudaki et al. (2024) to fit the collegiate consumption context. This 5-item scale covers intentions to purchase both tangible goods and experiential services over the next 6 months. Sample items include: “In the next six months, I intend to purchase high-quality professional sports equipment or apparel” and “I am willing to pay for a gym membership, sports training classes, or sporting event tickets.” The Cronbach’s *α* was 0.89, and the CR was 0.88.

### 3.3 Data analysis strategy

Data processing and model testing were conducted using SPSS 27.0 and AMOS 26.0. The analytical procedure involved four steps:

Data Pre-processing: Missing data across the tracking waves were handled using Full Information Maximum Likelihood (FIML) estimation.

Bias and Validity Checks: Harman’s single-factor test was performed to screen for Common Method Bias (CMB). A Confirmatory Factor Analysis (CFA) was executed to assess standardized factor loadings, Average Variance Extracted (AVE), and Composite Reliability (CR), thereby validating the convergent and discriminant validity of the measurement model.

Descriptive Statistics: Means, standard deviations, and Pearson correlation matrices for the core variables were calculated.

Longitudinal Serial Mediation Testing: A Structural Equation Model (SEM) was constructed to test the time-lagged main effects. Subsequently, controlling for gender, age, and university affiliation, a bias-corrected non-parametric percentile Bootstrapping method (with 5,000 resamples) was utilized to generate 95% Confidence Intervals (CI). This rigorously tested the serial mediating effects of “Value Internalization” and “Exercise Identity.” Mediation was considered statistically significant if the 95% CI did not include zero.

The three-wave autoregressive cross-lagged panel design adopted here offers several methodological strengths that bolster the basis for causal inference. First, by including autoregressive paths for each construct, the model isolates true incremental change in mediators and outcome from baseline stability, mitigating concerns about historical inertia and ensuring that the estimated cross-lagged effects represent developmental change rather than between-wave consistency. Second, the temporal ordering of predictors (T1), mediators (T2), and outcomes (T3) establishes the directional precedence required for mediation analysis, addressing a core limitation of cross-sectional designs in which the temporal relations among variables are confounded. Third, controlling for prior levels of the endogenous variables substantially attenuates threats from unobserved time-invariant heterogeneity and from reverse causality, two recognized sources of endogeneity in survey-based behavioral research. Although this design does not eliminate every potential source of bias (see Section 5.3), it provides a markedly stronger basis for inference than the cross-sectional designs that have predominated in prior work on sport policy and consumer behavior (Table 1).

**Table 1:** Demographic characteristics of participants (*N* = 652).

Variables	Categories	n	%
Gender	Male	345	52.9
	Female	307	47.1
University region	Central (Shanxi Institute of Energy)	318	48.8
	Eastern (Zhejiang Univ. of Finance & Economics)	334	51.2
Age	≤ 18 years	100	15.3
	19–20 years	380	58.3
	≥ 21 years	172	26.4
Grade Level	Freshman	202	31.0
	Sophomore	235	36.0
	Junior	168	25.8
	Senior	47	7.2
Monthly living expenses	≤ 1,500 RMB	142	21.8
	1,500–2,500 RMB	326	50.0
	≥ 2,500 RMB	184	28.2

## 4 Data analysis and results

### 4.1 Common method bias and measurement model validation

Although the present study employed a three-wave longitudinal design spanning 6 months, which provides procedural separation between predictor and outcome measurements and serves as a remedy against common method bias (CMB), all measures relied on participant self-report. Statistical procedures were therefore conducted to further evaluate potential CMB. Harman’s single-factor test was performed, and an unrotated principal component analysis extracted four factors with eigenvalues greater than 1. The first principal factor accounted for 31.4% of the total variance, below the conventional 40% threshold often used as an indicator of substantive CMB.

It is important to note, however, that recent methodological work has questioned the diagnostic sensitivity of Harman’s test as a stand-alone indicator. Howard et al. (2024), in a systematic examination of published management studies, demonstrated that Harman’s test does not reliably discriminate between research designs with varying degrees of method bias and cautioned against treating it as definitive evidence of CMB absence. The present study therefore relied principally on the procedural safeguards inherent to the three-wave design—temporal separation of predictors and outcomes, attention-check items, and identifier-based wave matching—to attenuate transient mood, consistency-motif, and demand-characteristic effects. Residual concerns associated with self-reported data are discussed in the limitations section.

To further validate the construct validity of the core variables (i.e., 12 latent constructs comprising sports policy attitude, value internalization, exercise identity, and sport consumption intention across the three time points), a CFA was performed in AMOS 26.0 using maximum likelihood estimation. The hypothesized four-factor measurement model showed excellent fit: *χ^2^*/*df* = 2.15, comparative fit index (CFI) = 0.962, Tucker-Lewis index (TLI) = 0.954, root mean square error of approximation (RMSEA) = 0.042, and standardized root mean square residual (SRMR) = 0.038. All standardized factor loadings ranged from 0.72 to 0.89 and were significant at *p* < 0.001. The CR of each construct exceeded the recommended 0.80 threshold, and all AVE values surpassed the 0.50 benchmark, supporting the convergent and discriminant validity of the measurement scales.

### 4.2 Descriptive statistics and correlations

Tables 2, 3 present the means, standard deviations, and Pearson correlation coefficients for the core variables. Descriptively, Value Internalization at T3 yielded the highest mean score (*M* = 3.95), reflecting a strong and progressive cognitive endorsement of sports values over time. Conversely, Sport Consumption Intention at T1 scored relatively lower (*M* = 3.65), aligning with the psychological principle that translating cognitive values into high-cost financial commitments is inherently challenging and requires a developmental psychological process.

**Table 2:** Descriptive statistics and reliability of main variables across time points.

Time point	Variable	*M*	*SD*	Cronbach’s *α*	CR
T1	Policy Attitude	3.85	0.72	0.88	0.89
	Value Internalization	3.90	0.80	0.90	0.91
	Exercise Identity	3.72	0.84	0.88	0.88
	Consumption Intention	3.65	0.88	0.91	0.92
T2	Policy Attitude	3.87	0.70	0.89	0.89
	Value Internalization	3.92	0.81	0.91	0.91
	Exercise Identity	3.76	0.85	0.89	0.90
	Consumption Intention	3.68	0.87	0.92	0.93
T3	Policy Attitude	3.88	0.71	0.88	0.89
	Value Internalization	3.95	0.79	0.92	0.92
	Exercise Identity	3.80	0.83	0.90	0.91
	Consumption Intention	3.72	0.86	0.91	0.92

*N =* 652. CR = Composite Reliability. All Cronbach’s α and CR values exceeded the recommended threshold of 0.80, indicating excellent internal consistency and reliability of the measurement models across all three time points.

**Table 3:** Longitudinal pearson correlation matrix of main variables across time points.

Variable	1	2	3	4	5	6	7	8	9	10	11	12
Attitude (T1)	1											
Internalization (T1)	0.42	1										
Identity (T1)	0.35	0.52	1									
Consumption (T1)	0.31	0.45	0.55	1								
Attitude (T2)	0.65	0.38	0.32	0.28	1							
Internalization (T2)	0.46	0.68	0.48	0.40	0.44	1						
Identity (T2)	0.38	0.55	0.66	0.50	0.36	0.54	1					
Consumption (T2)	0.34	0.48	0.58	0.64	0.32	0.46	0.56	1				
Attitude (T3)	0.58	0.35	0.30	0.25	0.63	0.37	0.31	0.27	1			
Internalization (T3)	0.41	0.56	0.44	0.38	0.45	0.65	0.47	0.39	0.43	1		
Identity (T3)	0.34	0.49	0.55	0.45	0.37	0.53	0.64	0.48	0.35	0.56	1	
Consumption (T3)	0.32	0.44	0.52	0.56	0.35	0.47	0.59	0.62	0.33	0.48	0.58	1

*N =* 652. Diagonal values are 1. For brevity, “Attitude” denotes Sports Policy Attitude, “Internalization” denotes Value Internalization, “Identity” denotes Exercise Identity, and “Consumption” denotes Sport Consumption Intention. The square root of the AVE for each construct was greater than its correlations with all other constructs, demonstrating adequate discriminant validity across all time points. All correlation coefficients presented in this table are statistically significant at the *p* < 0.001 level.

Correlation analyses provided solid preliminary support for the theoretical derivations. Specifically, T1 Policy Attitude was significantly and positively correlated with T3 Consumption Intention (*r* = 0.32, *p* < 0.001), laying the foundation for the main longitudinal effect. Furthermore, the inter-construct correlation coefficients ranged from 0.25 to 0.68, all of which were statistically significant at the *p* < 0.001 level and remained well below the multicollinearity warning threshold of 0.85.

A subsequent Variance Inflation Factor (VIF) check revealed that all VIF values for the predictor variables were below 3, well under the critical value of 10. This indicates that while the variables are logically and significantly related, they maintain sufficient statistical independence, effectively ruling out multicollinearity issues for the subsequent Structural Equation Modeling (SEM) and serial mediation tests.

### 4.3 Alternative model comparisons

Before testing the specific paths of the core hypotheses, this study evaluated the structural optimality of the proposed longitudinal serial mediation model (Model 1). In psychological and behavioral sciences, causal sequencing among variables often invites multiple theoretical interpretations. To empirically exclude alternative explanations, two theoretically plausible competing models were constructed based on literature reviews and compared against the baseline model regarding their goodness-of-fit.

Model 1 (Hypothesized Serial Mediation Model): Follows the “environmental perception → cognitive internalization → self-concept reconstruction → behavioral intention” logical sequence (T1 Attitude → T2 Value → T2 Identity → T3 Consumption).

Model 2 (Parallel Mediation Model): Based on stimulus–response assumptions, this model removes the causal path between value and identity, hypothesizing that T1 Policy Attitude simultaneously and independently triggers T2 Value Internalization and Exercise Identity, which then act as parallel mediators affecting T3 Consumption Intention.

Model 3 (Reverse Serial Mediation Model): Drawing on Self-perception Theory, this model explores potential reverse causality. It posits that individuals first adopt the “exerciser” label under policy influence (T2 Identity) and subsequently internalize sports values to maintain cognitive consistency (T2 Value), ultimately leading to consumption (T1 Attitude → T2 Identity → T2 Value → T3 Consumption).

As shown in Table 4, a comparison of fit indices and information criteria (AIC and BIC, where lower values indicate a better balance of fit and parsimony) clearly demonstrates that Model 1 achieved the optimal fit (*χ^2^*/*df* = 2.31, CFI = 0.958, TLI = 0.951, RMSEA = 0.045). In contrast, Model 2, which stripped away the internal causal mechanism, showed significantly deteriorated fit. Although Model 3 reached acceptable fit standards, its AIC (387.5) and BIC (470.8) were noticeably inferior to Model 1. This SEM comparison not only helps evaluate a plausible reverse-ordering explanation but also empirically corroborates the sequential logic integrating Self-Determination Theory and Identity Theory: individuals must first cognitively internalize values before they can reconstruct their identity at a deeper personality level.

**Table 4:** Fit indices of alternative structural models.

Models	*χ^2^*	*df*	*χ^2^*/*df*	CFI	TLI	RMSEA	AIC	BIC
Model 1 (Hypothesized Serial)	263.3	114	2.31	0.958	0.951	0.045	345.3	428.6
Model 2 (Parallel Mediation)	335.8	115	2.92	0.921	0.910	0.068	415.8	499.1
Model 3 (Reverse Serial)	305.5	114	2.68	0.935	0.926	0.059	387.5	470.8

### 4.4 Structural equation modeling and direct effects

**Figure 3:**
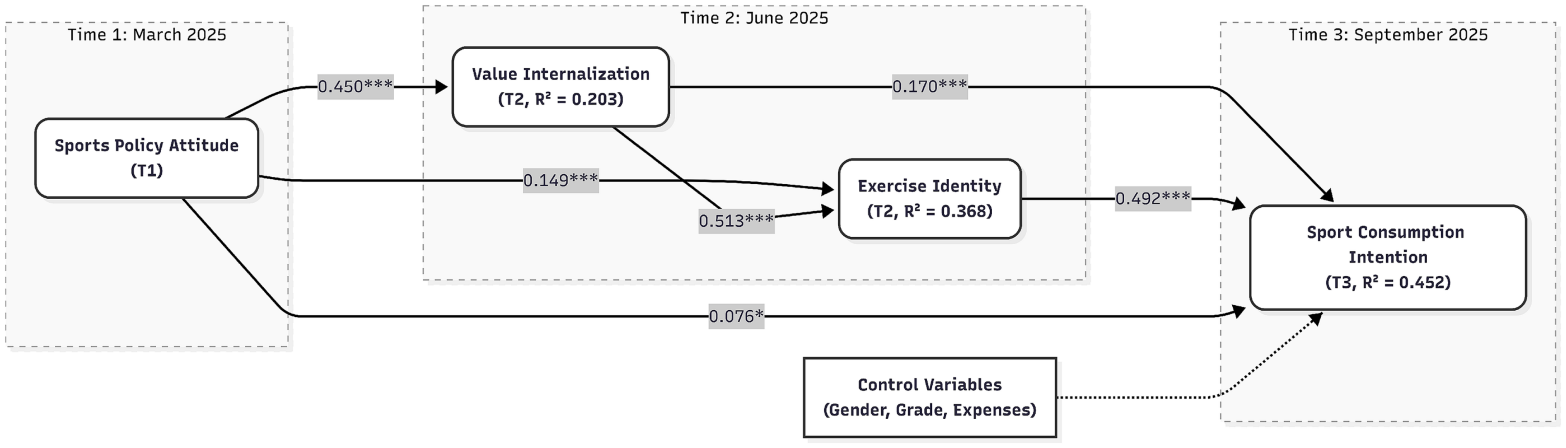
Final structural equation model with standardized path coefficients.

Having established the optimal structural configuration, Maximum Likelihood Estimation was utilized to estimate the overall path parameters. To ensure the purity of the causal relationships among core variables, three demographic covariates as well as the autoregressive paths were strictly controlled before estimating the cross-lagged main effects. Controlling for these autoregressive effects is a critical step in CLPM, as it ensures that the structural paths represent true developmental changes over time rather than mere historical inertia. The path coefficients for the control variables and core hypotheses are detailed in Figure 3 and Table 5.

**Table 5:** Unstandardized and standardized path coefficients of the structural model.

Structural paths	*B*	*SE*	*β*	CR*(t)*	*p*
Autoregressive paths					
T1 Value Internalization → T2 Value Internalization	0.582	0.041	0.560	14.22	< 0.001
T1 Exercise Identity → T2 Exercise Identity	0.551	0.040	0.530	13.80	< 0.001
T2 Consumption Intention → T3 Consumption Intention	0.443	0.043	0.420	10.15	< 0.001
Core cross-lagged paths (main effects)					
T1 Policy Attitude → T2 Value Internalization	0.506	0.040	0.450	12.65	< 0.001
T1 Policy Attitude → T2 Exercise Identity	0.176	0.040	0.149	4.40	< 0.001
T2 Value Internalization → T2 Exercise Identity	0.538	0.040	0.513	13.45	< 0.001
T2 Value Internalization → T3 Consumption Intention	0.166	0.035	0.170	4.74	< 0.001
T2 Exercise Identity → T3 Consumption Intention	0.457	0.035	0.492	13.05	< 0.001
T1 Policy Attitude → T3 Consumption Intention (Direct Effect)	0.083	0.035	0.076	2.37	0.018
Control variables paths					
Gender → T3 Consumption Intention	−0.126	0.063	−0.080	−2.00	0.045
Grade Level → T3 Consumption Intention	0.015	0.027	0.020	0.55	0.582
Living Expenses → T3 Consumption Intention	0.134	0.041	0.120	3.26	0.001

Gender is a dummy variable (1 = Male, 2 = Female). B = unstandardized coefficient; SE = standard error; β = standardized coefficient. Autoregressive paths were included to control for the baseline levels of the endogenous variables, ensuring that the core cross-lagged paths represent true incremental developmental changes over time.

The results regarding the covariates indicated that monthly living expenses significantly and positively predicted T3 Consumption Intention (*β* = 0.120, *p* = 0.001), which aligns with the economic rationale that resource endowment dictates purchasing power. The effect of gender on consumption intention was marginally significant (*β* = −0.080, *p* = 0.045), suggesting that males exhibited a slightly higher propensity for sports consumption than females. Grade level differences were non-significant (*p* > 0.05).

After isolating the interference of these confounders, the SEM path coefficients unveiled the dynamic interactions among the core variables. The model demonstrated robust explanatory power, accounting for 20.3% of the variance (*R^2^*) in Value Internalization, 36.8% in Exercise Identity, and 45.2% in Sport Consumption Intention—highly desirable levels of explanatory variance in longitudinal behavioral research.

Specifically, T1 Policy Attitude exhibited strong longitudinal predictive power on T2 Value Internalization (*β* = 0.450, *p* < 0.001) and T2 Exercise Identity (*β* = 0.149, *p* < 0.001). T2 Value Internalization substantially facilitated the formation of T2 Exercise Identity (*β* = 0.513, *p* < 0.001) and significantly enhanced T3 Consumption Intention (*β* = 0.170, *p* < 0.001). Ultimately, T2 Exercise Identity emerged as the strongest proximal driver of T3 Consumption Intention (*β* = 0.492, *p* < 0.001).

Particular attention must be paid to the main effect path: In the baseline model excluding mediators, the total effect of T1 Policy Attitude on T3 Consumption Intention was highly significant (*β* = 0.340, *p* < 0.001). However, upon introducing Value Internalization and Exercise Identity, the direct effect of T1 Policy Attitude on T3 Consumption Intention plummeted to *β* = 0.076. Although it retained marginal statistical significance (*p* = 0.018), this sharp attenuation in effect size clearly indicates the presence of a partial mediation mechanism. This result supports H_1_ and implies that macro-level policies can rarely bypass individual psychological processing to directly generate consumption; their efficacy relies overwhelmingly on mediating transformative mechanisms.

### 4.5 Serial mediation analysis (bootstrap test)

To further quantify and rigorously test the hypothesized mediation pathways (H_2_, H_3_, H_4_), a bias-corrected non-parametric percentile Bootstrap resampling method (5,000 resamples) was applied to estimate the confidence intervals (CI) of the indirect effects. A specific mediation path is considered statistically significant if the 95% CI does not encompass zero. The results and the proportion of mediation for each path are detailed in Table 6.

**Table 6:** Bootstrap results for the serial mediation effects and proportion of mediation.

Mediating pathways	*β*	Boot *SE*	95% CI Lower	95% CI Upper	Proportion	Result
Total indirect effect	0.264	0.028	0.211	0.320	100%	Significant
Ind 1: T1 Att → T2 Val → T3 Int (H_2_)	0.077	0.017	0.045	0.112	29.17%	Supported
Ind 2: T1 Att → T2 Id → T3 Int (H_3_)	0.073	0.018	0.038	0.109	27.65%	Supported
Ind 3: T1 Att → T2 Val → T2 Id → T3 Int (H_4_)	0.114	0.019	0.079	0.155	43.18%	Supported

The data in Table 6 provide precise quantitative evidence to unlock the “psychological black box” between policy perception and consumer behavior. The total indirect effect reached 0.264, with a 95% CI of [0.211, 0.320], excluding zero. Breaking down the specific paths:

- The independent mediating role of Value Internalization (H_2_): Path Ind 1 was significant (*β* = 0.077, 95% CI [0.045, 0.112]), contributing to 29.17% of the total indirect effect. This indicates that external policy drivers can indeed translate into partial consumption intention by enabling students to “recognize the benefits of sports,” supporting H_2_.
- The independent mediating role of Exercise Identity (H_3_): Path Ind 2 was significant (*β* = 0.073, 95% CI [0.038, 0.109]), accounting for 27.65% of the indirect effect. This suggests that the campus sports atmosphere fostered by policies can prompt some students to adopt the “exerciser” persona directly, triggering consumption intentions via the need for self-verification, supporting H_3_.
- The serial mediating role from Cognition to Identity (H4): The core sequential path Ind 3 (T1 Attitude → T2 Value → T2 Identity → T3 Consumption) exhibited the largest indirect effect (*β* = 0.114, 95% CI [0.079, 0.155]). This serial path accounted for the largest share (43.18%) of the total indirect effect.

The relative magnitudes of these effects (43.18% > 29.17% > 27.65%) carry theoretical implications for understanding the policy-to-consumption pathway. The pattern indicates that relying on cognitive evaluation alone (internalizing values) or on identity formation in isolation (assuming the exerciser role) is associated with comparatively weaker indirect effects on consumption intention. The largest indirect effect operates through the sequential pathway in which policy attitudes first shape cognitive assimilation of sport-related values, which in turn supports the consolidation of an exerciser identity, ultimately translating into sustained sport consumption intention. This pattern provides longitudinal evidence consistent with H_4_.

## 5 Discussion

### 5.1 Discussion of main findings

Drawing on a three-wave longitudinal dataset spanning 6 months, this study examined the time-lagged impact of college students’ sports policy attitudes on their sport consumption intention, and unpacked the sequential mediating mechanisms of value internalization and exercise identity. The findings extend prior work in three substantive directions.

First, the study provides an empirical refinement of “policy determinism” perspectives on sport behavior. The data indicate that a positive sports policy attitude (T1) significantly predicts consumption intention (T3), with a total effect of *β* = 0.340. This result is consistent with the perspective in sport economics that macro-institutional support and structural opportunities constitute important antecedents of individuals’ sport-related economic decisions (Downward et al., 2014). When the mediating variables were incorporated into the longitudinal model, however, the direct effect of policy attitude attenuated substantially to *β* = 0.076 and retained only marginal statistical significance. This pattern of partial mediation suggests that top-down policy directives rarely bypass individual psychological processing to directly generate micro-level consumption behavior. Policy efficacy appears to depend on the psychological transformation mechanisms operating at the level of the individual (Hagger and Chatzisarantis, 2016), and policy promulgation alone is unlikely to translate into market activity without these intermediate psychological processes.

Second, the findings clarify the functional differences between cognitive assimilation and self-concept reconstruction in driving sport consumption. The independent mediation analyses supported both the value internalization pathway (H_2_) and the exercise identity pathway (H_3_). Consistent with meta-analytic evidence in the SDT-exercise literature (Teixeira et al., 2012), value internalization—reflecting a shift from extrinsic to identified regulation—was associated with stronger consumption intention, accounting for 29.17% of the indirect effect. This result is also consistent with the proposition advanced by Funk et al. (2012) that autonomously regulated motivation underlies sustained engagement among sport consumers. The identity-based pathway contributed 27.65% of the indirect effect, in line with prior demonstrations that a strong physically active identity functions as a stable behavioral anchor (Strachan et al., 2010) and that identity-laden involvement organizes sustained consumption-related behaviors among sport participants (Beaton et al., 2011). Together, these results suggest that recognizing the value of sport (“sport is good”) and identifying with sport (“I am an exerciser”) operate as distinct, complementary motivational sources of sport consumption intention.

The third and most theoretically informative finding concerns the serial transformation from cognition to identity (H_4_). Prior cross-sectional work has often treated values and identity as parallel variables or has not specified their causal ordering. Through alternative model comparisons, the present study not only addressed reverse-causality concerns but also showed that the cognitive internalization → identity reconstruction serial pathway accounted for the largest share of the indirect effect (43.18%). This pattern provides longitudinal evidence consistent with the Trans-Contextual Model (Hagger and Chatzisarantis, 2016): macro-environmental cues such as policy attitudes appear to first shape an individual’s autonomous belief system before these beliefs crystallize into a stable identity structure. Within the sport consumption context, value internalization may be understood as the cognitive foundation upon which identity is built, while exercise identity functions as the load-bearing wall through which value translates into sustained, tangible economic engagement. Without value internalization, identity claims may lack ideological grounding; without identity, internalized values may not translate into sustained financial commitment. This sequential pathway delineates a coherent trajectory from external environment to internal cognition, to core self-concept, and ultimately to market behavior.

### 5.2 Theoretical and practical contributions

This study makes contributions in both theoretical development and managerial practice.

Theoretical contributions. First, the study addresses methodological limitations in prior research and establishes a clearer basis for causal inference. Earlier work has relied predominantly on cross-sectional data, leaving the relationship between policy environments, psychological mechanisms, and consumption behavior open to challenges of reverse causality. Through a three-wave cross-lagged design and alternative model comparisons, the present study provides longitudinal evidence consistent with the proposed sequence in which policy environments shape psychology and psychology drives consumption, contributing to the relatively limited body of longitudinal evidence in this field. Second, the study integrates the motivational dimension of Self-Determination Theory with the personality dimension of Identity Theory, suggesting that internalization functions as a precursor to identity formation in the sport consumption context. This integration offers an account of how macro-environmental signals translate into micro-level behavioral commitments, complementing the developmental logic of the Psychological Continuum Model (Funk et al., 2008; Funk and James, 2001) by anchoring its developmental stages to specific psychological mechanisms.

Practical contributions. For universities and policymakers, the findings suggest a shift in emphasis from behavioral discipline to identity awakening. Because exercise identity emerged as a proximal driver of consumption intention, university sport departments may benefit from moving beyond traditional check-in-based or attendance-based management approaches toward the cultivation of an autonomy-supportive campus sport ecosystem in which students can develop and consolidate an exerciser identity through sustained social interaction and meaningful participation. For the sport industry, the findings suggest that consumer motives among emerging adult consumers may be increasingly oriented toward identity expression alongside functional satisfaction. Marketing strategies that emphasize the identity-symbolic attributes of sport products—through community-building, meaningful brand narratives, and social platforms that allow consumers to enact and validate an exerciser identity—may complement traditional appeals to product functionality. These implications should be interpreted in light of the study’s limitations discussed below.

### 5.3 Limitations and future directions

Despite the rigorous design, several limitations of the present study should be acknowledged, each of which suggests productive avenues for future research.

First, regarding the ecological representativeness of the sample, longitudinal attrition constrained the study to two representative universities in central and eastern China. Although the cross-regional and cross-disciplinary sampling strategy mitigates some concerns about institutional homogeneity, future research using multi-center, large-sample national stratified random sampling would allow tests of cross-group invariance of the serial mediation model across students with different socioeconomic backgrounds.

Second, regarding the operationalization of the dependent variable, the present study measured consumption intention rather than actual expenditure. Although intention is a robust proximal predictor of behavior, the translation from intention to realized consumption is moderated by factors such as impulse control, objective budget constraints, and unanticipated situational influences. Future mixed-methods research that integrates objective transaction data—for example, from campus electronic payment platforms—would yield more direct evidence of the policy-to-consumption pathway.

Third, regarding the duration of follow-up, the six-month observation window was sufficient to capture the psychological transitions of interest, but longer panel studies spanning the full undergraduate trajectory or extending into early career stages would be needed to examine the developmental stability and life-course evolution of exercise identity, particularly during major life transitions.

Fourth, regarding the modeling approach, we adopted the traditional autoregressive cross-lagged panel model (CLPM), which has been the analytical convention in much existing longitudinal sport psychology research. Hamaker et al. (2015) and Mulder and Hamaker (2021) have raised the methodologically important concern that the traditional CLPM does not separate within-person fluctuations from stable between-person differences, and that the random-intercept CLPM (RI-CLPM) addresses this issue by explicitly modeling time-invariant individual heterogeneity. We retained the traditional CLPM in the present study for two reasons. First, our theoretical interest centers on between-person differences in how policy attitudes shape downstream consumption trajectories—a question for which the traditional CLPM remains informative. Second, RI-CLPM places stringent demands on the number of waves and on within-person variance, which our three-wave design only marginally satisfies. Future research with four or more measurement occasions should re-examine the proposed serial mechanism using RI-CLPM and related dynamic structural equation models to disentangle within- and between-person processes more rigorously.

Fifth, regarding the operationalization of value internalization, we measured this construct using the Identified Regulation subscale of the BREQ (Markland and Tobin, 2004). Although identified regulation marks the autonomous threshold of the OIT internalization continuum and is widely adopted in exercise-motivation research, it does not encompass the full spectrum of internalization, which also includes introjected and integrated regulation. Future research could employ the full BREQ-3 (Wilson et al., 2006) to examine whether the serial pathway documented here generalizes across the broader internalization continuum and whether different regulatory styles exhibit differentiated downstream effects on sport consumption behavior.

Sixth, with respect to common method bias, all measures relied on participant self-report. Although the three-wave longitudinal design provides procedural separation of predictors and outcomes, and Harman’s single-factor test did not indicate a dominant single factor, recent methodological work (Howard et al., 2024) has questioned the diagnostic sensitivity of Harman’s test. Future research could employ stronger designs—such as multi-source data collection or experimental policy manipulations—to provide more definitive safeguards against method-bias concerns.

## 6 Conclusion

Within the macro-context of China’s contemporary sport policy environment, college students’ sport consumption appears to reflect not a mechanical response to policy directives, but a process of psychological reconstruction. The longitudinal evidence presented here indicates that a positive sports policy perception predicts sport consumption intention through the sequential operation of value internalization and exercise identity. This cognition-to-identity transformation pathway offers an account of the psychological mechanisms underlying policy efficacy and provides theoretically grounded guidance for cultivating sustained sport engagement among emerging adults and for advancing the development of the sport consumer market.

## Data Availability

The raw data supporting the conclusions of this article will be made available by the authors, without undue reservation.
